# Dental caries and attendance to dental care in Finnish children with operated congenital heart disease. A practice based follow-up study

**DOI:** 10.1007/s40368-021-00603-8

**Published:** 2021-03-27

**Authors:** H. Karhumaa, E. Lämsä, H. Vähänikkilä, M. Blomqvist, T. Pätilä, V. Anttonen

**Affiliations:** 1 Department of Cariology, Endodontology and Pediatric Dentistry, University of Oulu, Oulu, Finland; 2 Medical Research Centre, Oulu University Hospital and University of Oulu, Oulu, Finland; 3 Infrastructure of Cohort Studies, University of Oulu, Oulu, Finland; 4 New Children’s Hospital, Helsinki University Central Hospital, Helsinki, Finland

**Keywords:** Children, Congenital heart disease, Dental caries, Dental attendance

## Abstract

**Purpose:**

Oral health of children with congenital heart disease (CHD) is of utmost importance. This study aimed to investigate the prevalence of dental caries and attendance to dental care in Finnish heart-operated CHD patients born in 1997–1999.

**Methods:**

The cohort of children born in 1997–1999 was selected using a national register on all heart-operated children in Finland. Gender, general health problems, diagnosis, type of the heart defect (shunting, stenotic and complex defects), and number of operations were available and included in the analyses. Dental records from primary health care were collected from municipalities with their permission. The data comprised of the number of dental examinations and data on caries status (dt, DT, dmft, DMFT) at the age of 7 (grade 1), 11 (grade 5) and 15 (grade 8) years and at the most recent examination. The control group consisted of dental data on patients born in 1997–1999 provided by the City of Oulu, Finland (*n* = 3356).

**Results:**

Oral patient records of 215/570 children were obtained. The difference between the defect types was statistically significant both for DT (*p* = 0.046) and DMFT (*p* = 0.009) at the age of 15 (grade 8). The prevalence of caries did not differ between the study population and the controls. High present and past caries experiences were not associated with higher number of visits to oral health care, especially to oral hygienist, or with oral health promotion. National obligations concerning dental visits were not implemented in all municipalities.

**Conclusion:**

There seems to be a need for oral health promotion and preventive means implemented by oral hygienists among those with CHD.

## Introduction

Congenital heart disease (CHD) is the most common developmental disorder of a single human organ. The incidence of any congenital heart disease is approximately 75–80/1,000 live births. The presence of heart defects is very similar in different populations (Hoffman and Kaplan 2002; Øyen et al. 2009; Dolk et al. 2011; Leirgul et al. 2014).

Classification of CHDs can be based on the characteristics of blood flow in the heart. The first type comprises shunting defects: ventricular septal defect (VSD), atrial septal defect (ASD), atrioventricular septal defect (AVSD), and patent ductus arteriosus (PDA). The second type consists of stenotic defects: aortic stenosis (AS), pulmonal stenosis (PS), and coarctation of aorta (CoA). The third type comprises complex defects: tetralogy of Fallot (TOF), transposition of great arteries (TGA), and hypoplastic left heart syndrome (HLHS). These ten types make up 90% of the heart defects. HLHS is an example of univentricular heart (UVH) defects. A patient can also have several types of heart defects simultaneously (Thiene and Frescura 2010). The most common CHDs can also be classified as cyanotic and acyanotic defects. In a cyanotic defect, the blood oxygen level is lower due to shunting between pulmonary and systemic circulation. Studies on oral health of CHD children often use the latter classification (Pimentel et al. 2013).

Several studies around the world have shown that the oral health of children with CHD is poorer compared to healthy ones. Consequently, the number of decayed, missed or filled teeth due to caries (dmft and DMFT indices describing caries prevalence) is often higher in children with CHD than in controls (Hallett et al 1992; Pollard and Curzon 1992; Stecksén-Blicks et al. 2004; Rosén et al. 2010; Ali et al. 2017; Frank et al. 2019). Furthermore, caries prevalence has been higher in patients with cyanotic defects compared to those with acyanotic defects (Pimentel et al. 2013). On the other hand, Oliver et al. (2018) reported that high caries prevalence in CHD children is associated with low socio-economic status, presence of other illnesses and enamel defects, but not with the type or severity of the heart disease.

Oral health behaviors of the children with heart disease may also be poorer than those of the controls (Suvarna et al. 2011; Ali et al. 2016, 2017). Due to poor oral hygiene, several endocarditis-inducing bacteria have been found in the oral cavity of children with CHD (Ali et al. 2016; Saunders and Roberts 1997). Indeed, children with CHD are at risk for infective endocarditis (Baltimore et al. 2015). Therefore, the American Heart Association (AHA) recently concluded that the focus should be on maintaining good oral hygiene, preventing gingival and dental diseases, and ensuring access to routine dental care for children at risk for infective endocarditis (IE). Cases of IE from oral bacterial pathogens in children most likely result from exposure to relatively frequent, low-grade bacteremia from these activities of daily living (Baltimore et al. 2015).

Dental attendance and oral hygiene may not be a priority when a child is severely ill and frequently hospitalised, as is the case with children with CHD. Children with CHD drop out of the dental care system more often than healthy ones (Ali et al. 2016; Saunders and Roberts 1997).

Dietary habits in children with CHD can be poor due to small and frequent meals (Suvarna et al. 2011; Hansson et al. 2012), indicated by increased saliva *Lactobacilli* counts (Pourmoghaddas et al. 2018). Frequent energy intake is commonly recommended for children with CHD.

All Finnish children are entitled to and attend municipal dental healthcare free of charge until they turn 18 years. Children are recommended to have dental examination by a dental professional at least when they are 1–2, 3–4, 5–6 years old, and examinations preferably by dentist in the 1st grade (7 years), 5th grade (11 years) and 8th grade (15 years). However, the municipalities have wide autonomy to organize children’s dental health care i.e., it is not defined which dental professional carries out the examinations. Practically all children attend municipal dental health care and respective index data based on their patient records are available for research with the permission of the register keeper or the municipality (Finnish Ministry of Justice 2011).

There is little information on oral conditions of this marginal group in Finland. Therefore, this study aims to investigate the prevalence of dental caries in association with the type of CHD and number of operations in children born 1997–1999. Another aim is to investigate attendance to dental care, both to oral hygienist and dentist. The third aim is to compare caries prevalence between the present study population and general population at the age of 7, 11 and 15 years.

## Materials and methods

Data were obtained using the surgical ProCardio patient registry of the New Children’s Hospital, Helsinki University Central Hospital, Finland. This registry includes all children who have required a heart operation at any age in Finland. Most of children were operated in infancy. A registry search was performed to identify CHD operated children born in 1997–1999. From these children, those surviving up to 18 years were selected (*n* = 570). The place of residence of the children was acquired from the Finnish National Population Register Center. All dental records of the children who had attended primary health care in only one municipality during their lifetime were requested from the local municipal health authorities. Finally, copies of all dental files of 215 (37.7%) patients were received from 124 Finnish municipalities (at present, the total number of Finnish municipalities is 310).

General health information of the participants was provided by ProCardio: date of birth, gender, type of heart defect [ventricular septal defect (VSD), atrial septal defect (ASD), atrioventricular septal defect (AVSD), patent ductus arteriosus (PDA), aortic stenosis (AS), pulmonal stenosis (PS), coarctation of aorta (CoA), tetralogy of Fallot (TOF), transposition of great arteries (TGA), univentricular heart defect (UVH)], dates of heart operations, and other illnesses (no other illness/syndrome/endocrinologic disease/asthma/neurologic disease/premature birth/other).

Dental history as well as information on dental attendance up to the age of 18 was collected from the patient documents obtained from the municipalities. The following information was recorded: number of dental examinations (*n*, date), number of dietary and oral hygiene counseling sessions/education (dietary or brushing education registered in the patient records), as well as all recorded dt/dmft or DT/DMFT indices. To compare caries prevalence with the general population, data were provided by the City of Oulu, Finland, on caries status (dt, dmft, DT, DMFT as well as dt + DT and dmft + DMFT) at the age of 7 (grade 1), 11 (grade 5) and 15 (grade 8) on children born 1997–1999 (*n* = 3576).

## Statistics

The study population was described as frequencies and proportions. In the analyses, the heart conditions were categorised according to the type of the heart defect (shunting, stenotic and complex). Association of the last mean dt/DT ja dmft/DMFT values before the person turned 18 with the heart condition and number of operations as well as with the number of dental visits were analyzed, considering the patients’ gender and other diseases. In the analyses, cross tabulation, and for testing statistical significance between the groups, Chi square test was used. Differences between the groups were considered statistically significant with *p* values < 0.05. Kruskal–Wallis and ANOVA tests were used to compare the means of caries indices between heart defect types, operations and between CHD children and healthy ones. All analyses were conducted using SPSS version 24.0 (Chicago, Illinois, USA).

## Ethics

The Finnish ProCardio database/register includes the data of all heart-operated children in Finland and has been used in previous studies (Sairanen et al. 2005; Raissadati et al. 2015). Parents have given permission to use the patient data in scientific research. The Helsinki University Hospital District has given a favorable ethical opinion on the study (165/13/03/03/2012). The Northern Ostrobothnia Hospital District gave their permission for this register study (248/2017).

We collected data of CHD children born in 1997–1999 and operated at least once in their childhood living in different parts of the country. Residential history of the study population was obtained from the national Population Register Center; with that information, patient records were collected from children’s primary health care centers. According to Finnish legislation, because only register information (indices, procedures) was collected, this was done with the permission of the register keeper (in this case, municipalities).

All analyses were carried out without any information on personal identification.

## Results

The research group was slightly dominated by boys (55.6% vs. 44.4%). The participants were 19–21 years old in 2018 when data were collected. Shunting defects were the most common heart defects, followed by complex ones. VSD was the most common defect, followed by PDA and CoA. Specifically, VSD and CoA were more prevalent among boys than girls (Table 1).

**Table 1:** Distribution of participants’ according to congenital heart disease and gender

Defect type *n* (%)	Type of heart disease	Boys *n* (%)	Girls *n* (%)	Total *n* (%)
Shunting 113 (52.8)	VSD(ventricular septal defect)	32 (68.1)	15 (31.9)	47 (22.0)
	ASD (atrial septal defect)	8 (34.8)	15 (65.2)	23 (10.7)
	AVSD (atrioventricular septal defect)	5 (50.0)	5 (50.0)	10 (4.7)
	PDA (patent ductus arteriosus)	18 (54.5)	15 (45.5)	33 (15.4)
Stenotic 32 (15.0)	AS (aortic stenosis)	3 (75.0)	1 (25.0)	4 (1.9)
	CoA (coarctation of aorta)	18 (69.2)	8 (30.8)	26 (12.1)
	PS (pulmonal stenosis)	1 (50.0)	1 (50.0)	2 (0.9)
Complex 48 (22.4)	UVH (univentricular heart defect)	6 (54.5)	5 (45.5)	11 (5.1)
	TOF (tetralogy of Fallot)	10 (43.5)	13 (56.5)	23 (10.7)
	TGA (transposition of great arteries)	7 (50.0)	7 (50.0)	14 (6.5)
Other 21 (9.8)	Other	11 (52.4)	10 47.6)	21 (9.8)
	Total	119 (55.6)	95 (44.4)	214 (100.0)

Of the study population, 17.8% had a syndrome; most commonly Down syndrome, followed by Catch22 and Turner syndrome. Asthma (15.9%) and neurologic disorders were also common (6.5%). The prevalence of the heart defects was statistically significantly associated with the presence of other diseases (*p* = 0.004); i.e., of those with syndromes, 42.1% had VSD, 21.1% had AVSD, and 18.4% had TOF. Children with PDA and VSD commonly had asthma (26.5% and 20.6%, respectively). Again, neurological disorders were commonly associated with PDA (35.7%). Children born with UVH often had two or more diseases (27.3%).

The difference between the defect types was statistically significant both for DT (*p* = 0.046) and DMFT (*p* = 0.009) at 15 years age; DT/DMFT values were the highest for children with ASD and TOF. The best situation was for children with AVSD and UVH in the age of 11 years and 15 years. As for the latest DMFT, the difference between defect types was also statistically significant (*p* = 0.006) (Table 2).

**Table 2:** Association of the dmft/DMFT indices with different heart defects

	11 years				15 years	
Type of heart defect	dt (SD)	dmft (SD)	DT (SD)	DMFT (SD)	DT (SD)	DMFT (SD)
VSD	0.3 (0,8)	3.6 (4.4)	0.5 (0.9)	1.0 (1.8)	0.7 (1.3)	1.9 (2.8)
ASD	0.4 (1.2)	2.8 (2.7)	0.6 (1,1)	1.6 (1.9)	1.9 (2.4)	3.7 (3.8)
AVSD	0.0 (0.0)	0.6 (1.3)	0.1 (0.3)	0.2 (0.7)	0.2 (0.7)	0.6 (1.7)
PDA	0.6 (0.4)	2.5 (3.6)	0.3 (0.6)	0.9 (1.5)	0.6 (1.3)	1.7 (2.2)
All shunting defects	0.2 (0.7)	2.9 (3.8)	0.4 (0.8)	1.1 (1.7)	0.9 (1.7)	2.1 (2.9)
AS	0.0 (0.0)	1.3 (2.5)	0.3 (0.5)	0.5 (0.6)	0.3 (0.5)	1.0 (0.8)
COA	0.0 (0.2)	1.8 (2.8)	0.1 (0.3)	0.7 (1.2)	0.4 (0.7)	1.8 (3.5)
PS	0.0 (0.0)	4.0 (5.7)	0.5 (0.7)	0.5 (0.7)	0.5 (0.7)	2.0 (1.4)
All stenotic defects	0.0 (0.2)	1.8 (2.9)	0.1 (0.3)	0.7 (1.1)	0.4 (0.7)	1.7 (3.2)
UVH	0.0 (0.0)	1.3 (2.1)	0.2 (0.4)	0.2 (0.4)	0.2 (0.6)	0.5 (1.0)
TOF	0.2 (0.5)	3.2 (5.1)	0.3 (0.7)	0.6 (1.1)	1.0 (1.2)	2.0 (2.6)
TGA	0.2 (0.4)	2.2 (2.9)	0.2 (0.6)	1.2 (1.9)	0.8 (1.3)	2.4 (2.6)
All complex defects	0.1 (0.4)	2.4 (4.0)	0.3 (0.6)	0.6 (1,3)	0.8 (1.1)	1.7 (2.4)
Other	0.5 (1.2)	2.3 (2.0)	0.2 (0.4)	0.8 (1.2)	0.3 (0.6)	1.0 (1.5)
Total	0.2 (0.7)	2.6 (3.6)	0.3 (0.7)	0.9 (1.5)	0.7 (1.4)	1.9 (2.8)
*P* values	0.353	0.349	0.610	0.482	0.046*	0.009*

The mean number of heart operations was 1.3 (SD 0.9, min 1, max 9) and mean age at operation was 1.9 years; However, the mean age at first operation was 1.1 years. The mean number of operations among children with complex CHD was 2.0 (SD 1.4, max 9), among those with shunting defects 1.1 (SD 0.3, max 3), and among those with stenotic defects 1.3 (SD 0.7, max 3). Caries status (dmft/DMFT) was slightly better among those operated two times or more than among those operated only once. Finnish children with CHD had similar dmft/DMFT values as their age-matched, healthy counterparts in the city of Oulu, Finland. (Table 3).

**Table 3:** Means of dt/DT and dmft/DMFT between CHD children and control group

		7 years	11 years	15 years	Last dental examination before 18 years
CHD children (*n* = 214)	dt/DT (SD)	0.8/0.2 (1.6/0.6)	0.2/0.3 (0.7/0.7)	− /0.7 (1.4)	0.3/1.0 (0.8/1.9)
Control (*n* = 3576)	dt/DT	0.8/0.2	0.1/0.6	− /1.2	
CHD children (*n* = 214)	dmft/DMFT (SD)	1.9/0.3 (3.2/0.8)	2.6/0.9 (3.6/1.5)	− /1.9 (2.8)	2.5/2.9 (3.5/3.9)
Control (*n* = 3570)	dmft/DMFT	− /0.2	− /1.3	− /2.7	
Heart operated once (*n* = 174)	dt/DT (SD)	0.9/0.2 (1.7/0.7)	0.2/0.4 (0.7/0.7)	− /0.7 (1.4)	0.3/1.0 (0.9/2.0)
Heart operated once (*n* = 174)	dmft/DMFT (SD)	2.2/0.3 (3.4/0.9)	2.8/1.0 (3.6/1.5)	− /2.0 (2.9)	2.72/3.1 (3.6/4.1)
Heart operated two or more times (*n* = 40)	dt/DT (SD)	0.3/0.0 (0.6/0.0)	0.1/0.1 (0.3/0.6)	− /0.7 (1.4)	0.2/1.0 (0.5/1.9)
Heart operated two or more times (*n* = 40)	dmft/DMFT (SD)	0.7/0.1 (1.5/0.5)	1.6/0.3 (3.1/0.9)	− /1.1 (1.9)	1.7/2.1 (3.3/3.0)

On average, CHD children had had 11.2 oral examinations: 7.9 (SD 3.7, min 0, max 19) visits to dentist and 3.3 (SD 3.1, min 0 max 15) to oral hygienist. Children with aorta stenosis (AS) had the most examinations (overall 14.8 times SD 4.6 min 8, max 18) which included 7.3 visits to dentist (SD 7.4, min 2, max 18) and 7.5 visits to oral hygienist (SD 6.5, min 0, max 15). Children with TGA had the least visits; overall 7.9 (SD 5.0): 6.1 visits to dentist (4.0, min 1, max 13) and 1.8 visits to oral hygienist (SD 2.1, min 0, max 7).

In the age of 15 years, CHD children with present restorative treatment need (dt > 0) had significantly fewer visits to dentist and oral hygienist and they also received less oral health promotion than the rest (Table 4). The same was true for oral health promotion when their DT > 0. Those with high caries experience in primary dentition (dmft ≥ 4) had received fluoride therapy but no other oral health promotion. Children with high past caries experience in permanent dentition (DMFT ≥ 4) had distinctly fewer visits to dentist and oral hygienist and less preventive means than the rest (Table 4). The number of oral examinations was not associated with the number of heart operations.

**Table 4:** Mean number of oral health care visits of children with CHD, classified by dt/dmft and DT/DMFT values in the 11 years age

	dt = 0	dt > 0	*p*	dmft = 0	dmft 1–3	dmft ≥ 4	*p*	DT = 0	DT > 0	*p*	DMFT = 0	DMFT 1–3	DMFT ≥ 4	*p*
Exams by dentist	8.3 (3.6)	6.1 (4.0)	0.001*	7.1 (3.3)	9.0 (3.5)	8.2 (4.2)	0.006*	8.2 (3.3)	7.3 (4.6)	0.080	8.1 (3.3)	8.9 (4.2)	5.9 (4.0)	0.003*
Exams by oral hygienist	3.4 (3.2)	2.3 (2.6)	0.055	3.7 (3.3)	3.1 (3.0)	2.8 (3.0)	0.136	3.3 (3.2)	3.1 (3.1)	0.672	3.4 (3.2)	3.9 (3.3)	1.6 (2.1)	0.001*
All dental exams	11.8 (4.0)	8.5 (5.2)	0.000*	10.9 (3.6)	12.1 (4.3)	11.0 (5.2)	0.293	11.5 (3.9)	10.3 (5.6)	0.110	11.5 (3.8)	12.8 (4.6)	7.6 (4.6)	0.000*
Dietary guidance	6.4 (4.3)	4.8 (4.2)	0.006*	6.5 (4.4)	6.4 (4.0)	5.6 (4.5)	0.168	6.5 (4.5)	5.1 (3.7)	0.028*	6.8 (4.6)	5.4 (3.5)	5.0 (3.9)	0.038*
Brushing guidance	8.0 (4.1)	6.1 (5.5)	0.000*	7.8 (4.4)	8.3 (3.8)	7.1 (4.7)	0.133	8.0 (4.3)	6.9 (4.8)	0.040*	8.1 (4.3)	7.6 (3.5)	6.3 (5.7)	0.017*
Extra fluoride	0.4 (0.5)	0.4 (0.5)	0.514	0.3 (0.5)	0.4 (0.5)	0.5 (0.5)	0.012*	0.4 (0.5)	0.3 (0.5)	0.111	0.4 (0.5)	0.4 (0.5)	0.4 (0.5)	0.601

## Discussion

Heart diseases were associated with male gender, general diseases and syndromes. Caries experience in permanent teeth, but not in primary dentition, was associated with the type of the heart defect. Those with ASD and TOF had the most caries experience in permanent dentition. There was no significant difference in caries status in different age groups between the study group and general population. Those who had caries did not have more visits to oral hygienist or get more oral health promotion rather the opposite.

The prevalence of congenital heart diseases is in accord with that in other countries (Hoffmann and Kaplan 2002; Leirgul et al. 2014). Here, CHD was more common among boys than girls, which is in line with a recent study from Finland (Raissadati et al. 2015). The numbers are contradictory to results from a recent survey in Norway where congenital heart diseases were more frequent in girls, with a birth prevalence of CHD of 13.6/1000 in girls compared to 13.1/1000 in boys (Leirgul et al. 2014). This may be due to the inclusion in this study of children with Down syndrome, which is more prevalent among boys than girls (Hoffman and Kaplan 2002). Again, Down syndrome is associated with CHD even if there is great variation between countries (Dolk et al. 2011).

The size of the study population here was somewhat bigger (*n* = 214) than in corresponding studies elsewhere (Ali et al. 2016,2017); Stecksén-Blicks et al. 2004; Tasioula et al. 2008). The bigger study population size is due to the practice-based nature of the present study. The study population comprised different parts of the country and was, therefore, representative according to residential factors. The Finnish health care system is publicly funded and equal to all citizens, and all children under 18 years are entitled to free dental care (Finnish ministry of Justice 2011). Therefore, data are available for a study like the present one, which is a definite strength. Practically all children attend public dental health care, which may make this study population homogeneous or decrease the effect of socio-economic factors shown to cause differences in oral health in other studies (Oliver et al. 2018; Frank et al. 2019), also among adults in Finland (Sakki et al. 1994). Heart operations are freely available for all children with CHD and the Finnish welfare system provides financial and practical support to families with diseased children. Heart operations are concentrated to Helsinki University children’s hospital; as a result, the national ProCardio registry was available for a study like the present one, which is another strength.

The data in this retrospective practice-based study are based on patient records from primary health care dentists. These have been found to be reliable (Hausen et al. 2001; Ljung et al. 2019). Due to the nature of this study, the outcome was not in any way affected by the activity of the participants; however, dental data and attendance in the past were still achieved. Requests were sent to each municipality. Small municipalities were the most active to respond to the request. Limited data from big cities may have influenced the results because oral health status has been shown to be influenced by the place of residence. In the study by Kämppi et al. (2013), oral health status was better in urban than in rural areas of Finland among men in their twenties. The relatively low response rate by municipalities and especially big cities is a shortcoming. Finnish current care guidelines for controlling caries propose that in children BW radiographs are indicated when at least one dentinal caries lesion is detected in clinical examination (Controlling Caries, Current Care Guidelines 2020). If this was the practice, could not monitored.

To have as comprehensive data, in analyses, data of the age groups 1st, 5th and 8th graders were selected. However, these time points seemed to be missing in many municipalities in this study, which raises concern for this patient group.

The control group comprised age-matched children from the city of Oulu, the number being about 3500. It would have been valuable to have controls from the same communities as the cases, but this was not possible. The information of place of residence was obtained from the registry of the National Population Register Center. Only children who had lived in the same community their whole childhood were included in the study population because collecting data from various communities would have been challenging.

There was no difference in the caries status between the CHD children and the controls, which is in line with a study from the UK (Tasioula et al. 2008). In their study, dmft/DMFT mean values were 1.6/0.8 for CHD children and 1.8/0.4 for the control group. The study included children aged 2–16 years (Tasioula et al. 2008). However, in Sweden, the situation was contradictory, when among children approximately 6.5 years of age (*n* = 41 in both groups), dmfs value was 5.2 for those with CHD compared to 2.2 in the control group (Stecksén-Blicks et al. 2004). In a study from Iran with 74 healthy and 68 children with CHD (mean age 7.4 yrs.), the DMFT was higher in CHD children (6.4) in comparison with healthy children (5.5) (Pourmoghaddas et al. 2018). The reason for these differences can only be speculated.

On average, the number of heart operations was 1.3 (SD. 0.9). Surprisingly, there was a trend that those with several operations had better caries status than the rest. The number of oral examinations and preventive care were not associated with the number of heart operations. More research is needed on the topic.

Current restorative treatment need was associated with fewer visits to oral health services per se. Past caries experience was positively associated with the number of visits to dentist but not to oral hygienist. The result indicates further need for preventive means among caries active children. The need for preventive means among caries active CHD children is well known (Sivertsen et al. 2018). There is too little awareness of preventative dental care and its importance among this patient population and their parents (Rai et al. 2009; Saunders and Roberts 1997). Oral hygienists should be involved in preventive work, which was not the case here.

## Conclusion

In the present study, the hypothesis that children with CHD would have more caries treatment need and history than their age-matched counterparts appeared not to be true. General national regulations concerning dental visits were not implemented in all municipalities, which is a concern for children with CHD. There seems to be more need for knowledge on the oral health of CHD children and for oral health promotion and preventive means implemented by oral hygienists especially among CHD children.

## Data Availability

Data are available from corresponding author per request.
